# Multi-Frequency Electro-Thermal Digital Model for Near-Field Electromagnetic Power Dissipation Mapping via Transient IR Thermography

**DOI:** 10.3390/s26196174

**Published:** 2026-09-29

**Authors:** Simona Miclaus, David Vatamanu, Ladislau Matekovits

**Affiliations:** 1 Department of Communications, IT & Cyber Defense, “Nicolae Balcescu” Land Forces Academy, 550170 Sibiu, Romania; 2 Department of Electronics and Telecommunications, Politecnico di Torino, 10129 Turin, Italy; ladislau.matekovits@polito.it; 3 Istituto di Elettronica e di Ingegneria dell’Informazione e delle Telecomunicazioni, National Research Council of Italy, 10129 Turin, Italy; 4 Department of Measurements and Optical Electronics, Politehnica University Timișoara, 300006 Timișoara, Romania

**Keywords:** electro-thermal digital model, transient IR thermography, near-field power dissipation, microstrip patch array, ITO thin-film transducer, finite-element thermal modeling

## Abstract

This paper presents a multi-frequency electro-thermal Digital Model framework for accurate mapping and prediction of near-field electromagnetic (EM) power dissipation and localized temperature rise in high-frequency planar microwave structures. The proposed methodology integrates 3D full-wave computational simulations in CST Studio Suite with non-invasive transient infrared (IR) thermography using an Indium Tin Oxide (ITO) coated Polyethylene Terephthalate (PET) thin-film transducer. A high-gain 4 × 4 microstrip patch antenna array operating nominally at 12.00 GHz serves as the experimental benchmark. Numerical and physical evaluations were conducted across five discrete operating frequencies (7.10, 8.05, 9.05, 11.14, and 12.00) GHz at near-field evaluation distances of 1 mm, 16 mm, and 29 mm under a continuous-wave microwave (MW) excitation over a 50 s exposure duration. Transient surface temperature dynamics were recorded using a calibrated Teledyne FLIR A700 LWIR radiometric sensor. Quantitative line profile validation yields an average maximum temperature rise error of 20.75%, an average spatial center shift of 2.70 mm, and an average spatial spread (FWHM) deviation of 21.29%. These moderate discrepancies are physically attributed to evaluating free-space electric field intensity (|E|^2^) within the numerical domain versus measuring resistive heat generation on the physical ITO film. The developed Digital Model framework provides a robust foundation for real-time thermal monitoring, localized hotspot suppression, and predictive thermal management in advanced phased arrays and high-power MW systems.

## 1. Introduction

Validating the spatial radiation characteristics and near-field dynamics of antenna systems remains one of the most resource-intensive and time-consuming phases in microwave (MW) engineering [1,2]. For decades, the gold standard for radiation pattern synthesis has relied on localized anechoic chambers and high-precision, probe-based mechanical near-field scanners [3,4]. Although highly accurate, these conventional methodologies represent a critical bottleneck in rapid prototyping cycles due to substantial capital costs, extended spatial acquisition times, and the requirement for specialized facilities. Consequently, there is an urgent demand for non-invasive, high-resolution diagnostic alternatives capable of capturing complex electromagnetic (EM) near-field distributions in real time while maintaining strict adherence to underlying physical principles [5,6,7].

To address these limitations, several wide-field and optical characterization techniques have been explored. Early implementations of visual EM field mapping utilized Liquid Crystal Sheets (LCS), which leverage temperature-dependent color transitions induced by RF absorption. However, LCS-based diagnostics exhibit a narrow dynamic range, non-linear thermal response, and substantial thermal hysteresis, rendering them inadequate for quantitative, high-fidelity Digital Twin synchronization [8,9,10]. Subsequent advancements introduced electro-optic (EO) sensors and micro-rectenna arrays, which minimize reactive field perturbations. Nevertheless, these systems inherently depend on point-by-point spatial rastering, thereby inheriting the time constraints of mechanical scanners and limiting spatial sampling density [11,12,13].

Recently, Infrared (IR) thermography has emerged as a promising wide-field diagnostic technique due to its capacity to capture the two-dimensional thermal footprint of an electromagnetic field within a single frame [14,15,16]. Prior research has demonstrated the utility of IR radiometric sensors for mapping surface current distributions on metallic structures and evaluating Specific Absorption Rate (SAR) profiles in lossy phantom media. Nonetheless, the majority of existing studies remain restricted to qualitative evaluations or are confined to far-field regimes, where planar wave fronts simplify spatial field dynamics [17,18].

A critical gap persists in leveraging quantitative IR thermography as a real-time validation bridge for a synchronized Digital Twin within the reactive and radiating near-field zones. In these complex regions, intricate spatial interference patterns are highly sensitive to the structural nuances of the radiator, such as multi-slot geometries and feeding network configurations.

This study addresses this gap by establishing a synchronized multiphysics Digital Model framework (as the first step to the final targeted Digital Twin) for near-field antenna characterization. Rather than treating numerical simulation and physical testing as isolated workflows, this methodology integrates high-resolution radiometric sensing with full-wave electro-thermal co-simulations. The foundational mechanism relies on capturing the transient thermal signature of the absorbed electromagnetic power density on an Indium Tin Oxide (ITO) coated Polyethylene Terephthalate (PET) thin film functioning as an EM-to-thermal transducer. A high-sensitivity radiometric thermal sensor (Teledyne FLIR A700) converts invisible near-field radiation into calibrated two-dimensional spatial temperature maps.

For experimental validation, a high-gain 4 × 4 microstrip patch antenna array operating nominally at 12.00 GHz (with secondary resonant responses evaluated at 7.10, 8.05, 9.05, and 11.14 GHz) was fabricated and fully characterized. By mapping complex field morphologies at near-field distances of 1 mm, 16 mm, and 29 mm, encompassing the reactive near-field and radiating near-field transition zones, this work demonstrates that transient thermal sensing provides a robust, highly efficient, and cost-effective alternative to probe-based scanning in anechoic environments.

## 2. Antenna Design, Fabrication, and Microwave Characterization

### 2.1. Topology and Geometrical Synthesis of the 4 × 4 Array

To serve as the physical benchmark for the electro-thermal Digital Model, a high-gain, planar 4 × 4 microstrip patch antenna array operating nominally at f_0_ = 12.00 GHz was designed and synthesized by the authors [19]. The radiating structure is implemented on a high-frequency Rogers RO4350B hydrocarbon ceramic laminate dielectric substrate (Rogers Corporation, Chandler, AZ, USA), characterized by a relative permittivity ε_r_ = 3.66, a substrate thickness h = 0.5 mm, and a dissipation factor tan δ = 0.0037. The foundational radiating element consists of a rectangular microstrip patch fed by a microstrip line. The initial resonant dimensions of the individual patch radiator—specifically its physical width (W) and length (L)—were derived using transmission line model formulations:

(1) $$W=\frac{c}{2f_{0}}\sqrt{\left(\frac{2}{\varepsilon_{r}+1}\right)}$$

where c represents the speed of light in free space. The effective dielectric constant (ε_eff_), which accounts for the fringing fields extending beyond the physical perimeter of the patch into the surrounding air region, is expressed as:

(2) $$\varepsilon_{eff}=\frac{\varepsilon_{r}+1}{2}+\frac{\varepsilon_{r}-1}{2}{\left(1+12\frac{h}{W}\right)}^{-\frac{1}{2}}$$

Due to fringing effects, the radiating patch appears electrically larger than its physical dimensions. The line extension length (ΔL) at each radiating edge is computed as:

(3) $$\Delta L=0.412\;h\frac{\left(\varepsilon_{eff}+0.3\right)\left(\frac{W}{h}+0.264\right)}{\left(\varepsilon_{eff}-0.258\right)\left(\frac{W}{h}+0.8\right)}$$

Consequently, the resonant physical length (L) required to satisfy the fundamental TM_10_ mode resonance condition is given by:

(4) $$L=\frac{c}{2f_{0}\sqrt{\varepsilon_{eff}}}-2\Delta L$$

To achieve high spatial directivity and facilitate distinct near-field hotspot formations, 16 resonant elements were arranged in a symmetric 4 × 4 planar matrix. Inter-element lattice spacings (d_x_ and d_y_) along the X- and Y-axes were constrained to $d_{x}=d_{y}\approx 0.6\lambda_{0}-0.7\lambda_{0}$ at 12 GHz, striking an optimal balance between minimizing mutual coupling and preventing the emergence of grating lobes in the visible space.

Corporate feeding topology was utilized to deliver equal-amplitude and equiphase excitation across all 16 patches. Impedance matching between the 50 Ω main feeding port and the multi-branch power splitters was accomplished using multi-stage quarter-wavelength (λ/4) microstrip transformers with characteristic impedances $Z_{T}$ calculated via:

(5) $$Z_{T}=\sqrt{Z_{in}Z_{L}}$$

where $Z_{in}$ and $Z_{L}$ denote the input impedance and branch load impedance, respectively. This configuration ensures uniform excitation across the array aperture, forming well-defined energy concentration nodes within the reactive near-field region.

### 2.2. Electromagnetic Full-Wave Simulation Strategy

**Figure 1:**
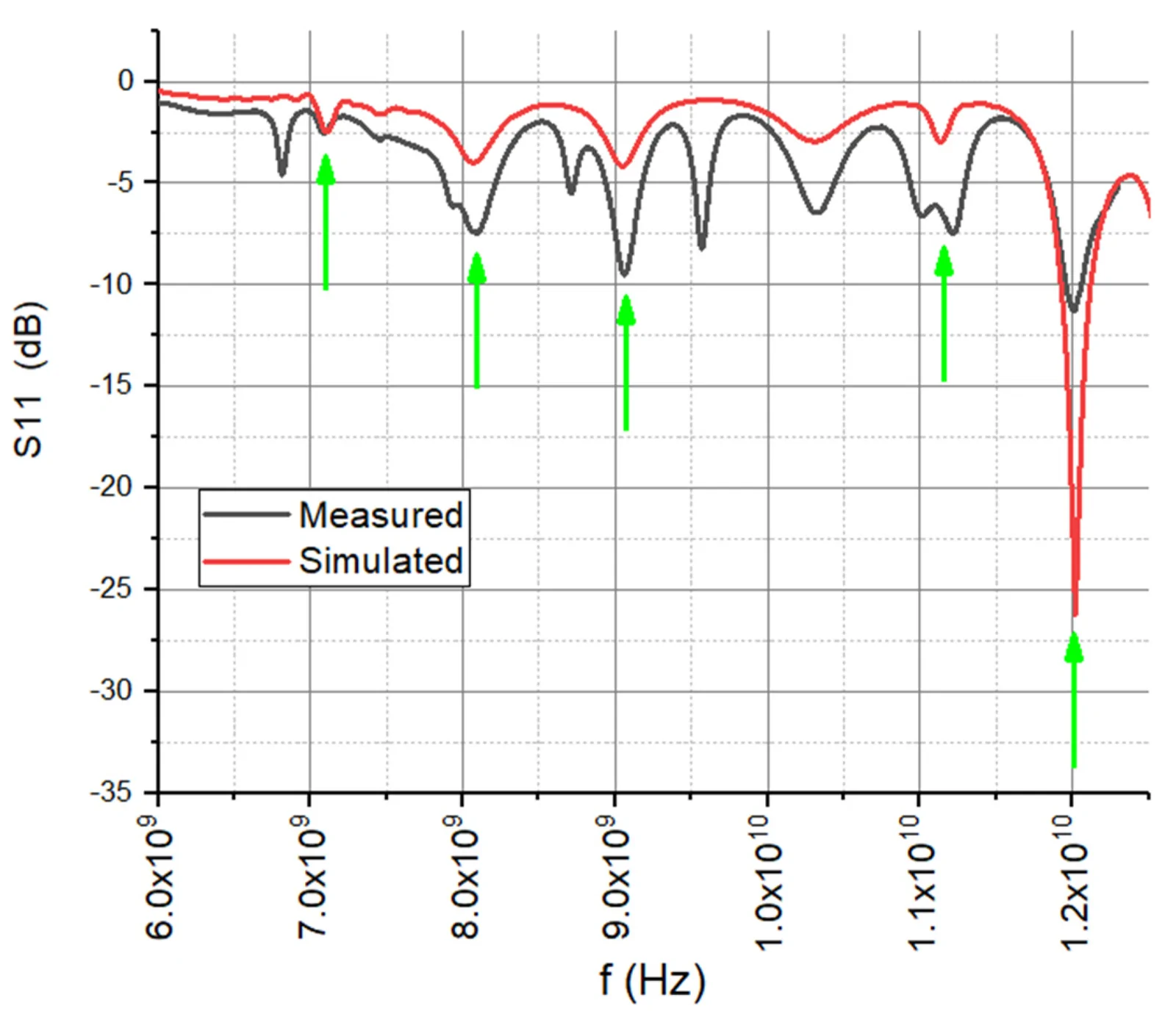
Reflection coefficient (S11) frequency response of the 4 × 4 patch array, comparing CST full-wave numerical simulation (red curve) with experimental vector network analyzer measurements (black curve). Green arrows indicate the five target operating frequencies evaluated in this study.

To evaluate the electro-thermal response across multiple operational regimes, full-wave 3D electromagnetic simulations were conducted in CST Studio Suite. While the baseline array geometry resonates at f_0_ = 12.00 GHz, the full-wave numerical strategy spans five discrete secondary-resonant excitation frequencies (f_0_ = 12 GHz, f_1_ = 7.10 GHz; f_2_ = 8.05 GHz; f_3_ = 9.05 GHz; f_4_ = 11.14 GHz) to capture frequency-dependent return loss, spatial field distributions, and power dissipation patterns. The reflection coefficient S11 frequency response of the fabricated array was evaluated against 3D full-wave CST simulations, as illustrated in Figure 1. Good quantitative agreement is observed between predicted and vector network analyzer (VNA) measured resonant notches.

The numerical setup employs the Finite Integration Technique (FIT) in the time domain on a non-uniform hexahedral mesh. Local mesh refinement (Δz = 0.05 mm, Δx, Δy = 0.1 mm) was applied around the feed lines, impedance transformers, and patch edges to resolve high current gradients and field concentrations accurately.

The simulation environment was configured with the following parameters:

- Boundary Conditions: Absorbing Perfectly Matched Layers (PML) open boundaries placed at λ/4 away from the array perimeter to model free-space radiation without reflections.
- Port Excitation: A 50 Ω waveguide port at the primary array input, driven by a transient pulse to compute broadband S-parameters and field profiles across all 5 target frequencies.
- Material Properties: Rogers RO4350B substrate (ε_r_ = 3.66, h = 0.5 mm, tan δ = 0.0037) and lossy Copper traces (t = 35 μm, σ_Cu_= 5.8 × 10^7^ S/m) accounting for dispersion and skin-effect losses.

**Figure 2:**
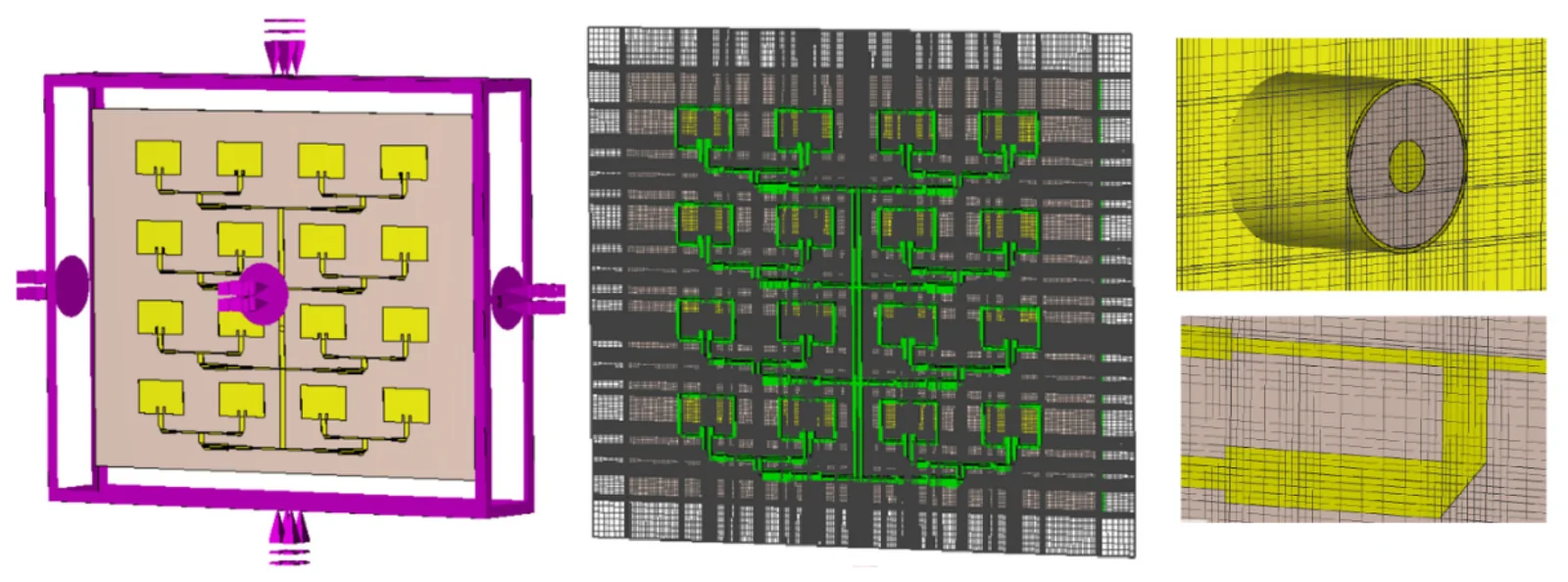
Numerical computational domain and discretization setup in CST Studio Suite: 3D CAD model of the 4 × 4 array, open PML boundary conditions, localized hexahedral mesh refinement along trace edges and 50 Ω waveguide port model.

To ensure high numerical precision, the simulation environment was structured as shown in Figure 2. The model incorporates open PML boundary conditions placed λ/4 away from the array perimeter, driven by a 50 Ω waveguide port, with local hexahedral mesh refinement concentrated around the feeding lines, impedance transformers, and patch edges.

For each of the 5 selected frequencies, the full-wave solver extracts the spatial distribution of the volumetric EM power loss density Q_em_ (W/m^3^), which is a sum of the two terms below:

(6) $$Q_{dielectric}=\frac{1}{2}\omega \varepsilon_{0}\varepsilon_{r}{tan\delta \left|E\right|}^{2}$$

(7) $$Q_{ohmic,s}=\frac{1}{2t}\sqrt{\frac{\omega \mu_{0}}{2\sigma_{Cu}}}{\left|J_{s}\right|}^{2}$$

Equation (7) represents the surface ohmic loss Q_ohmic,s_ (W/m^2^) across the metallic traces, whereas Equation (6) provides the volumetric dielectric loss Q_dielectric_ (W/m^3^) within the substrate. To couple these correctly into the 3D transient heat conduction equation (Equation (8)) as a unified volumetric heat source (W/m^3^), the surface power loss must be divided by the effective thickness of the Copper traces (t = 35 μm). In CST Studio Suite’s multi-physics co-simulation workflow, surface losses and dielectric losses are mapped directly onto their respective discretized mesh elements as equivalent volumetric heat generation rates (W/m^3^) via the transient solver’s built-in loss-to-thermal interface.

**Figure 3:**
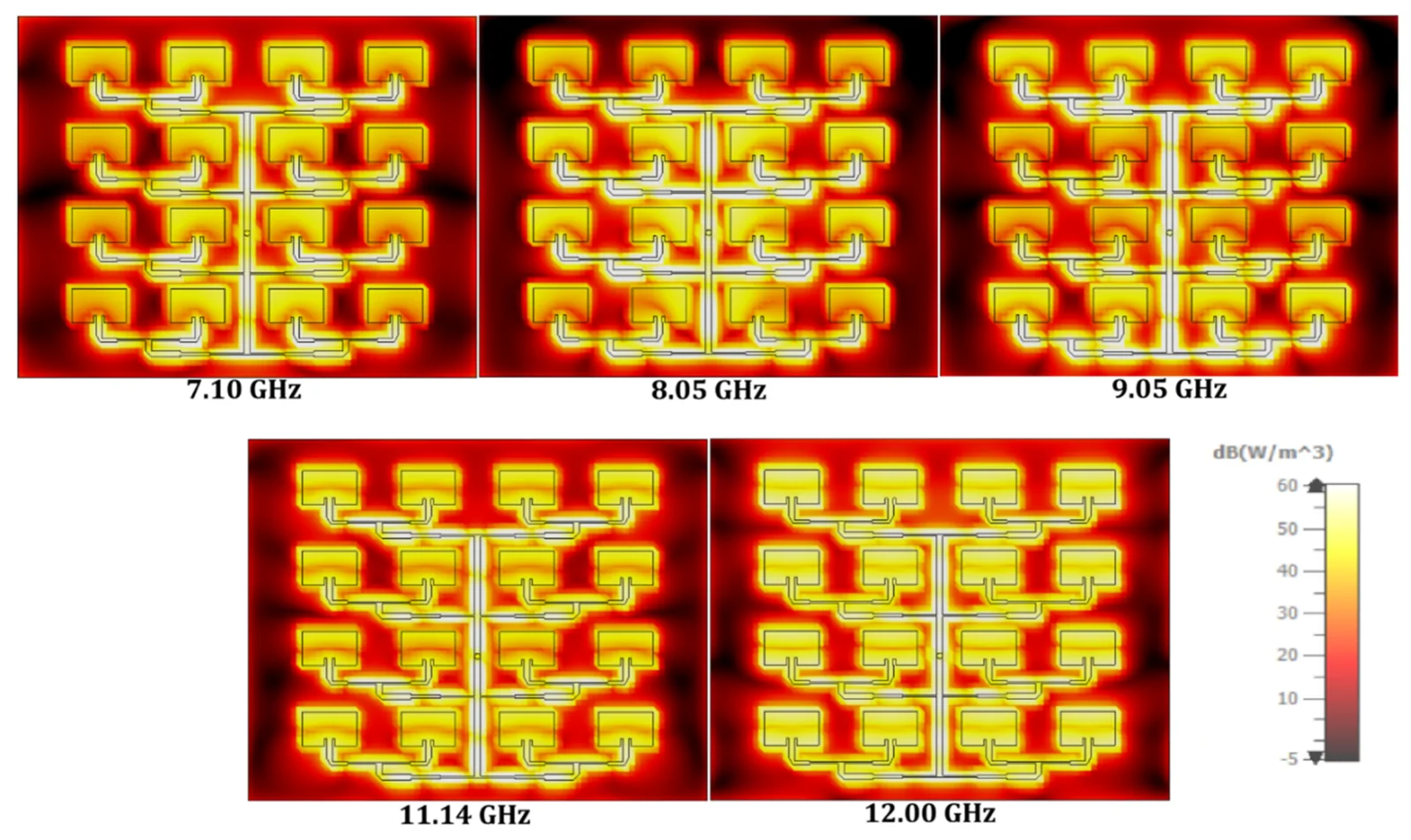
Computed spatial power loss density (Q_em_) distributions across the array aperture and lossy substrate for all five operating frequencies, illustrating the highlighted frequency-dependent thermal excitation nodes.

To identify the electromagnetic loss mechanisms, the spatial power loss density (Q_em_) distributions across the copper trace metallization and Rogers RO4350B substrate were extracted at all five operating frequencies (Figure 3).

These frequency-specific loss distributions serve as the distributed thermal heat source inputs for the multi-frequency electro-thermal Digital Model.

### 2.3. Thermal Digital Model Formulation and Boundary Conditions

To accurately model the transient spatio-temporal temperature rise induced by high-frequency power dissipation, a 3D finite-element thermal Digital Model was formulated. The thermal domain directly mirrors the physical dimensions and material layer stack-up of the 4 × 4 microstrip patch array (12.00 GHz) evaluated in the full-wave electromagnetic analysis.

The spatial and temporal evolution of the temperature field T(r, t) within the solid domains (dielectric substrate and metallic cladding) is governed by the 3D transient heat conduction equation:

(8) $$\rho C_{p}\frac{\partial T(r,t)}{\partial t}-\nabla (k\nabla T\left(r,t\right))=Q_{em}\left(r,f_{i}\right)$$

where ρ is the material mass density (kg/m^3^), C_p_ is the specific heat capacity (J/(kg·K)), k is the thermal conductivity (W/(m·K)), Q_em_(r, f_i_) is the spatial electromagnetic loss density (W/m^3^) imported directly from the CST full-wave solver at each specific operating frequency f_i_ (i = 0…4).

The accuracy of the thermal Digital Model depends on precise thermophysical parameters assigned to each domain:

- Rogers RO4350B substrate: isotropic thermal conductivity k_dielectric_ = 0.62 W/(m·K), density ρ = 1860 kg/m^3^, and specific heat C_p, diel_ = 1000 J/(kg·K).
- Copper traces (t = 35 μm): thermal conductivity k_Cu_ = 401 W/(m·K), density ρ = 8960 kg/m^3^, and specific heat C_p, Cu_ = 385 J/(kg·K).

The thermal domain models heat dissipation from the external surfaces to the ambient air through combined free convection and thermal radiation:

(9) $$-k\frac{\partial T}{\partial n}=h_{conv}(T_{s}-T_{amb})+\varepsilon_{em}\sigma_{SB}\left(T_{s}^{4}-T_{amb}^{4}\right)$$

where n represents the outward normal unit vector to the exterior boundary surfaces, T_s_ = T(r, t) at surface—is the local surface temperature, T_amb_ = 298.15 K is the ambient room temperature, h_conv_ is the natural convection heat transfer coefficient, set to 10 W/(m^2^·K) for still air horizontal surfaces, σ_SB_ = 5.6704 × 10^−8^ W/(m^2^·K^4^) is the Stefan-Boltzmann constant, ε_em_ is the surface thermal emissivity (≈0.05 for polished copper, and ≈0.92 for high-emissivity dielectric regions—to match IR thermography requirements).

The initial state across the entire array structure at time t = 0 is assumed to be in thermal equilibrium with the environment:

(10) $$T\left(r,0\right)=T_{amb}=25.0\;{}^{\circ}C$$

**Figure 4:**
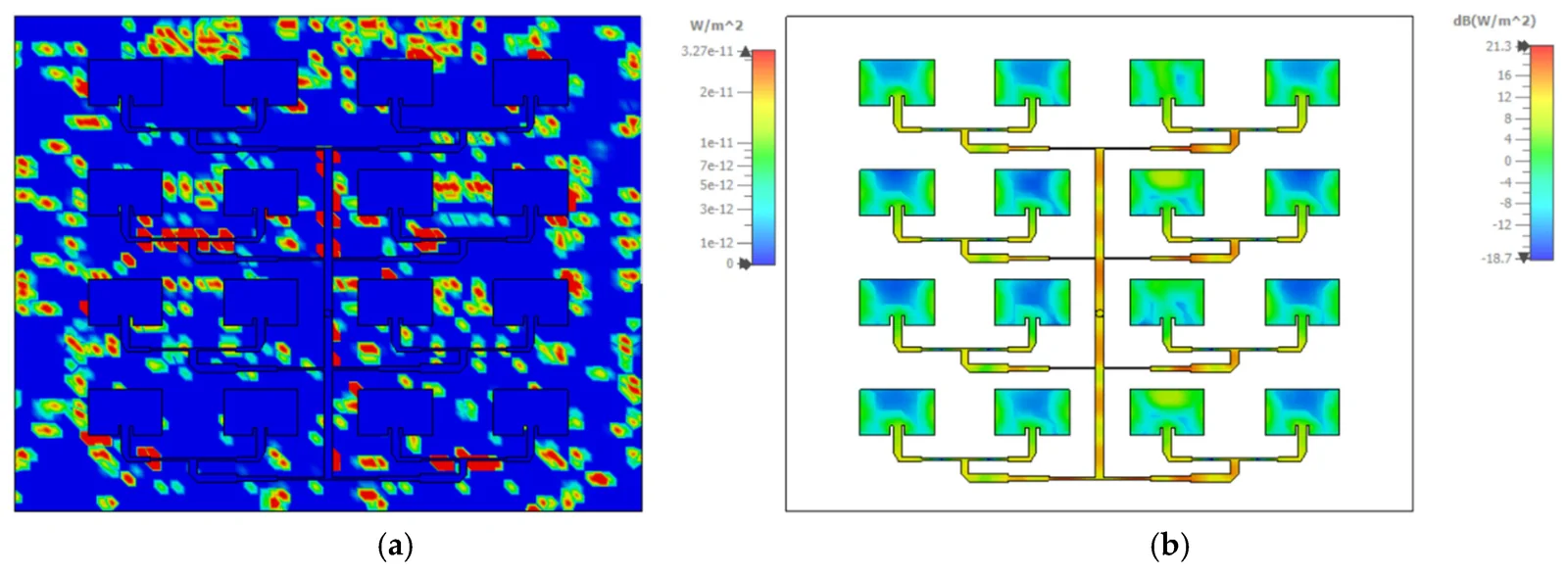
Numerical thermal simulation results after 50 s of MW excitation at 12 GHz: (**a**) internal heat flow density distribution within the dielectric substrate; (**b**) surface thermal energy losses across the conductor traces.

**Figure 5:**
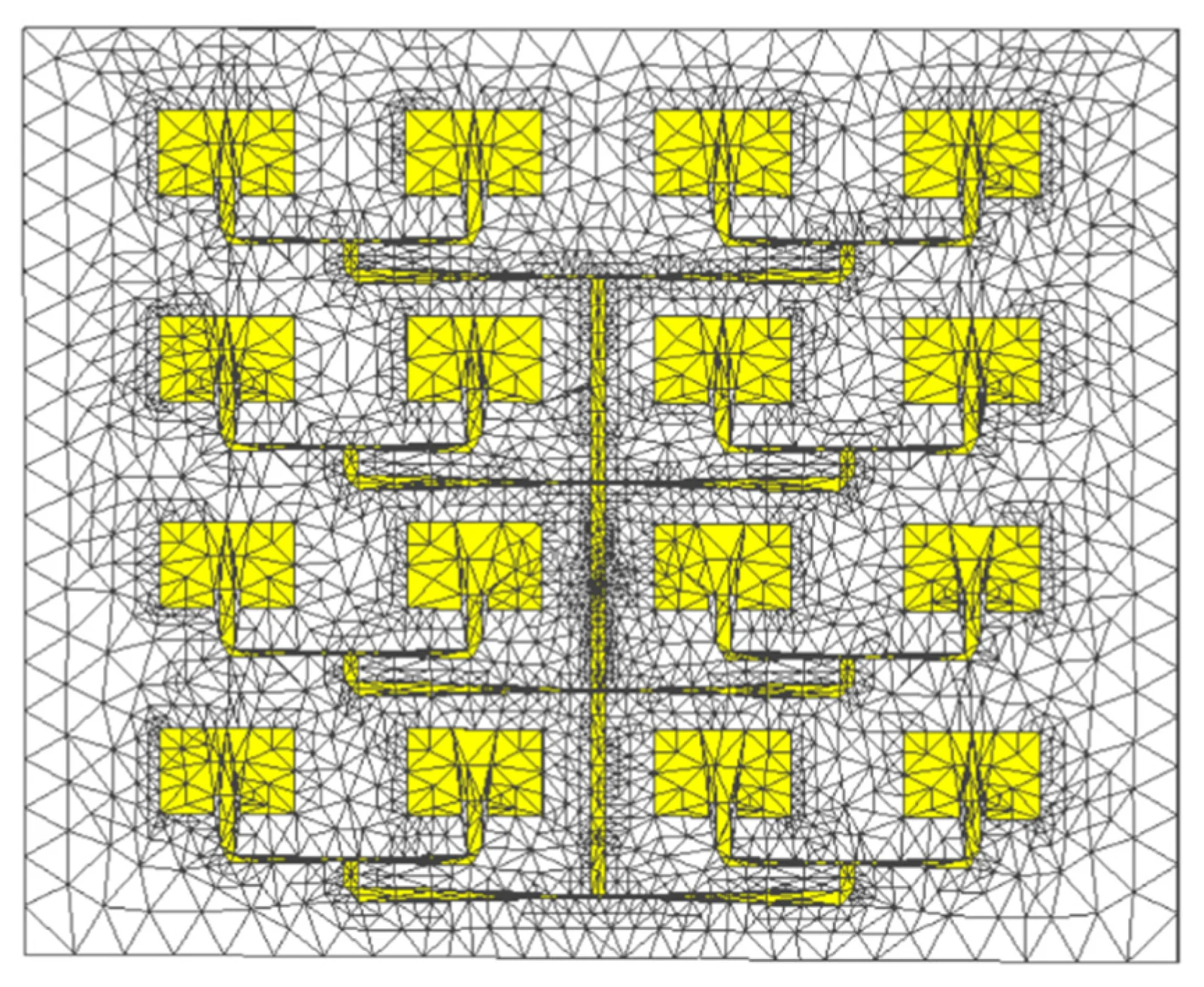
Finite-element thermal mesh discretization across the array geometry, highlighting local mesh refinement and continuum interface matching between the 35 μm thin copper metallization layer and the Rogers RO4350B substrate.

The transient heat flow density inside the dielectric substrate, illustrating the lateral thermal diffusion mechanisms governed by Equations (7) and (8), is outlined in Figure 4a, while the losses on the surface can be observed in Figure 4b. As shown in Figure 5, a continuous interface mesh matching was enforced between the 35 μm Copper metallization layer and the 0.5 mm Rogers RO4350B dielectric substrate for accurate simulations.

### 2.4. Transient IR Thermography Experimental Setup and Calibration

**Figure 6:**
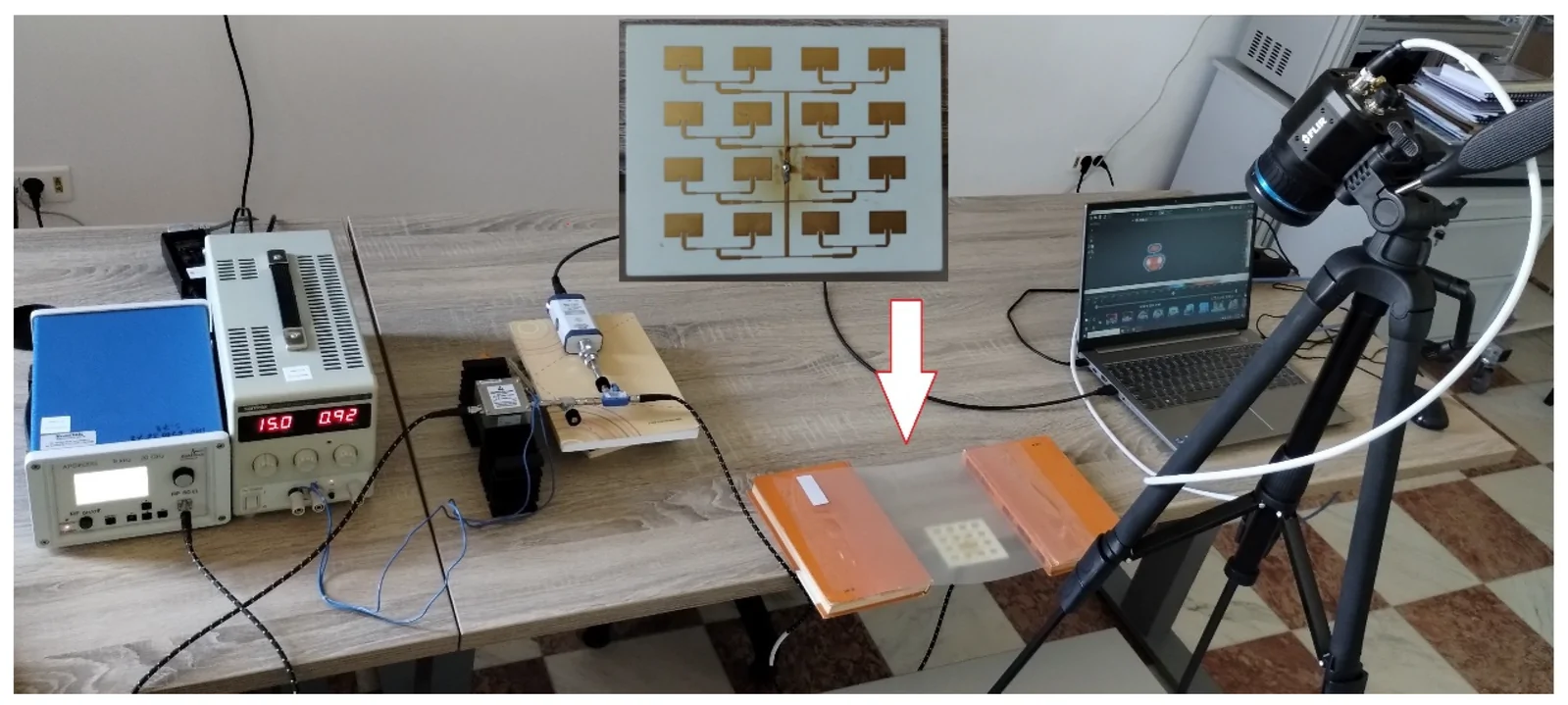
Experimental testbed layout for non-invasive transient IR thermography characterization, showing the continuous-wave (CW) microwave signal delivery chain, the 4 × 4 patch array positioned beneath the ITO/PET transducer thin film and the Teledyne FLIR A700 IR camera.

To validate the extremely high-frequency electro-thermal Digital Model across the five target frequencies, a non-invasive transient IR thermography testbed was established. The experimental setup (Figure 6) enables direct, real-time mapping of surface temperature variations induced by localized MW power dissipation without introducing field perturbations associated with physical metallic probes.

The experimental configuration consists of three primary subsystems:

- MW power generation and delivery: an AnaPico APSIN 20G signal generator (Ana Pico AG, Zurich, Switzerland) synthesized CW signals across the test band (7.10–12.00 GHz). The signal was amplified using a Mini-Circuits ZVE-3W-183+ amplifier (Mini-Circuits, Brooklyn, NY, USA) and routed through a directional coupler and circulator, to continuously monitor incident and reflected MW power; we used a R&S NRP18E power sensor (Rohde&Schwarz, Munich, Germany) to monitor the input power which was set at 2 W.
- Antenna and transducer solution: The 4 × 4 patch array antenna (enlarged above the arrow in the window of Figure 6) was positioned below an Indium Tin Oxide (ITO) coated Polyethylene Terephthalate (PET) thin film (Biotain Crystal Co., Limited, Longya, China) (observed as translucent film over the antenna in Figure 6), at three different distances: 1 mm, 16 mm and 29 mm; our choice of these distances was designed to systematically encompass the reactive near-field and the radiating near-field transition zones. At the nominal operating frequency of 12.00 GHz, the free-space wavelength, λ_0_, is approximately 25 mm; therefore, the selected distances can be quantitatively set as follows: (a) 1 mm (≈ 0.04 λ_0_)—is deep within the reactive near-field region, where evanescent waves dominate; (b) 16 mm (approx 0.64 λ_0_)—is situated in the radiating near-field (Fresnel region), capturing the transition where propagating waves begin to form but the radiation pattern is not yet fully synthesized; (c) 29 mm (approx 1.16 λ_0_)—is located in the outer boundary of the radiating near-field, moving toward the far-field (Fraunhofer) transition zone is approximately 25 mm. The film was used as an electromagnetic-to-thermal transducer. The film features a 13 nm thick ITO deposition with a sheet resistance of 300 Ω/sq. This specific sheet resistance achieves near-optimal impedance matching with the wave impedance of free space (Z_0_ ≈ 377 Ω). Due to this impedance match, the film acts as an excellent absorber/transducer that converts electromagnetic energy into heat without causing significant reflections back toward the radiating elements. Consequently, this setup allows for the spatial resolution of the electromagnetic field hotspot distribution without perturbing the resonant modes of the unit cells. The link between the free-space electric field distribution (|E|^2^) and the measured surface temperature rise (ΔT) is established through electromagnetic power absorption followed by Joule heating within the resistive ITO layer. Specifically, the incident high-frequency power density absorbed by the ultra-thin conductive film acts as a distributed volumetric/surface heat source $Q_{em}=\frac{1}{2}\sigma_{ITO}{\left|E_{tangent}\right|}^{2}$. This absorbed electromagnetic energy is subsequently governed by the 3D transient heat conduction equation, where the resulting temperature field scales proportionally with the local electromagnetic energy dissipation profile over the 50 s exposure window. Owing to the nanoscale thickness of the conductive layer, the transducer exhibits negligible thermal mass and minimal lateral heat diffusion, enabling rapid thermal response and localized heat dissipation via Joule losses. Furthermore, the high IR emissivity of the PET substrate combined with the low thermal conductivity of the underlying air-gap backing prevents unwanted thermal loading and spatial heat spreading, preserving the high spatial resolution of the near-field power dissipation maps. This setup allows sub-millimeter spatial resolution of the electromagnetic field hotspot distribution without perturbing the resonant modes of the unit cells. The three distances correspond to the reactive- and radiative near field of the antenna.
- Thermal Imaging System: The thermal distribution on the ITO film was captured using a Long-Wave Infrared (LWIR) radiometric sensor (Teledyne FLIR, Wilsonville, OR, USA) operating in the 7.5–14 μm spectral range. Thermal dynamics were recorded using a Teledyne FLIR A700 camera (thermal sensitivity of <0.05 K) enabled by Flir Reasearch Studio software v. 2025.01 (Figure 6).
- Environmental Isolation and Mounting: The physical prototype was placed on a low-loss wood support to minimize background thermal conduction losses and suppress parasitic MW reflections.

Accurate thermographic temperature measurement requires precise knowledge of the surface emissivity (ε_em_) across heterogeneous material regions (Copper traces vs. exposed dielectric substrate). Because polished copper exhibits low thermal emissivity (ε_Cu_ ≈ 0.05), raw thermograms can suffer from severe thermal reflections and inaccurate temperature readings.

To resolve this emissivity contrast, two calibration strategies were implemented:

- Emissivity Normalization Layer: A thin (approx. 15–20 μm height per applied pass), uniform layer of high-emissivity matte black coating (Graphit 33 spray—Kontakt Chemie, CRC Industries, Zele, Belgium) was applied to the front aperture, converting the surface into a uniform thermal emitter. The coating uniformity was verified dynamically using the IR camera while the prototype was on the isothermal heating plate (60.0 °C). By monitoring the surface without microwave excitation, we ensured the extracted digital pixel maps exhibited no spatial anomalies, guaranteeing a homogeneous emissivity coefficient across the entire aperture.
- Isothermal Reference Calibration: The prototype was placed on a temperature-controlled heating plate with embedded K-type thermocouples. The structure was heated uniformly from 25.0 to 60.0 °C without MW excitation. Digital emissivity maps were extracted by pixel-wise fitting of the IR camera raw digital counts against contact thermocouple readings.

For each of the five operating frequencies, the MW excitation was applied as a continuous step-function with a duration of 50s ON, followed by a cooling phase of 50 s OFF. The 50 s heating window provides a sufficient thermal signal-to-noise ratio while remaining short enough to limit spatial blurring caused by lateral heat diffusion through the dielectric substrate. Thermal frames were logged continuously at a frame rate of 16 Hz to capture the fast transient response at MW power onset.

## 3. Results and Validation of the Multi-Frequency Electro-Thermal Digital Model

To validate the high-frequency electro-thermal Digital Model, simulated surface temperature distributions T_sim_(r, t) were compared against calibrated IR thermography measurements T_meas_(r, t) under identical CW power excitation conditions. The analysis evaluates both steady-state surface thermal patterns and fast transient heating kinetics t_ON_ = 50 s across the five discrete operating frequencies (f_0_ to f_4_).

The physical transfer function linking the simulated electric field distribution to the thermal response relies on direct electromagnetic power conversion. Specifically, the tangential components of the electric field induce conduction currents within the lossy ITO resistive layer, generating a localized surface heat flux density (Q_em_ ~ |E|^2^). This absorbed energy dictates the transient temperature profile via Fourier’s heat conduction equation, providing the direct bridge between numerical electric field mapping and experimental IR thermography.

The spatial distribution of heat on the array surface exhibits a strong dependence on the MW excitation frequency f_i_, directly reflecting the frequency-dependent return loss S11 and internal power reflection:

- At main resonance (f_0_ = 12 GHz), the input MW energy is efficiently transferred and radiated across the 16 patches. Thermal dissipation is distributed symmetrically across the array aperture, with localized hot spots appearing along high-current microstrip transformer junctions and patch resonant edges.
- At secondary resonance frequencies (f_1_ to f_4_): Although these frequencies correspond to secondary resonance modes of the structure, their lower resonance amplitudes and higher return loss values (S11) cause a larger portion of the input power to be dissipated in the corporate feed network rather than radiated.

**Figure 7:**
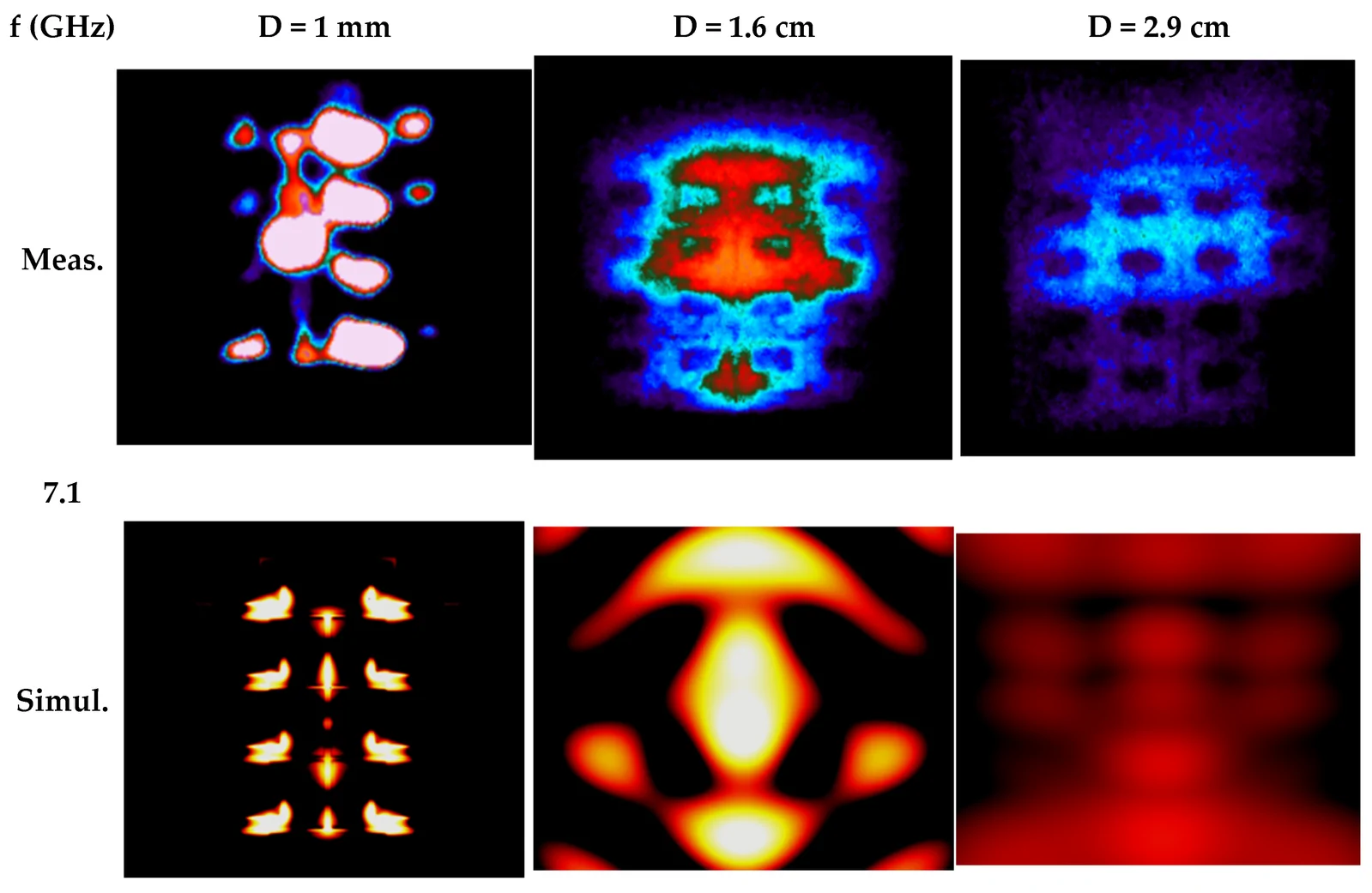

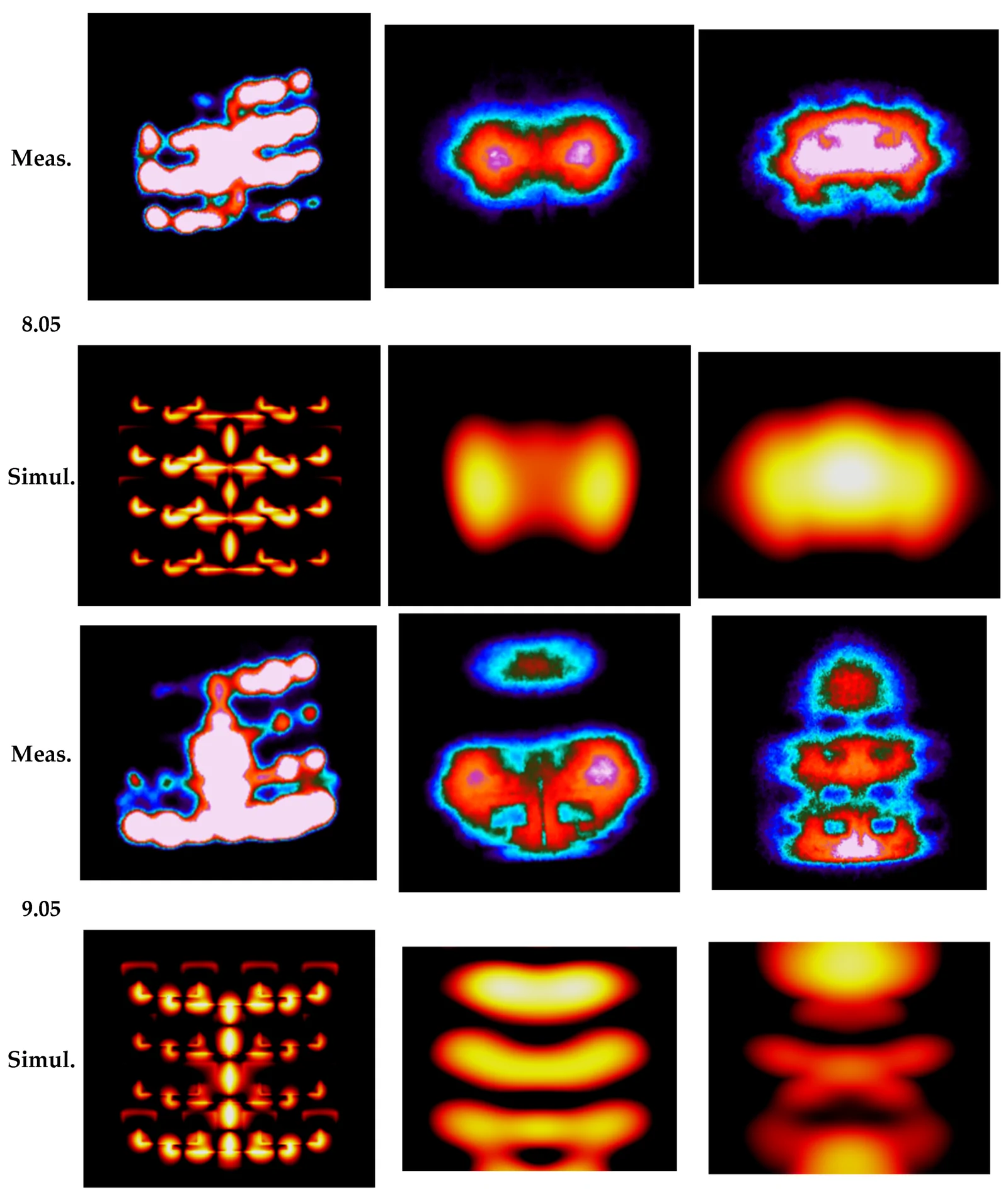

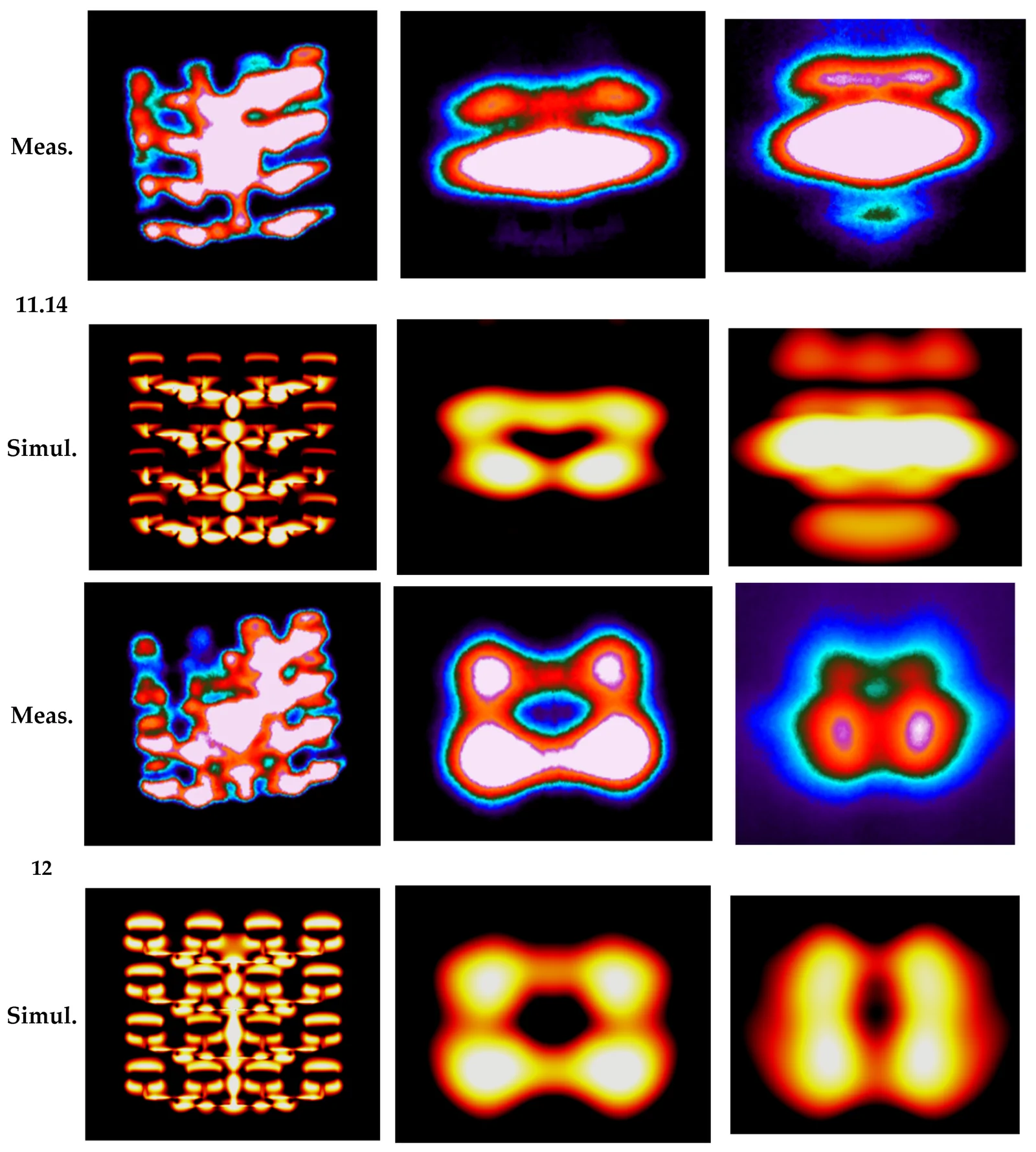
Qualitative and quantitative spatial pattern validation: comparison between calibrated experimental LWIR thermograms (top rows) and full-wave simulated power loss density distributions (bottom rows) across five excitation frequencies (7.10, 8.05, 9.05, 11.14, and 12.00 GHz) at three near-field evaluation planes situated at 1 mm, 16 mm, and 29 mm respectively).

As illustrated in Figure 7, the experimental spatial heat patterns recorded via LWIR thermography show strong spatial correlation with the simulated electromagnetic power loss density (Q_em_) maps across all evaluated frequencies and near-field planes at 1 mm, 16 mm, and 29 mm. In the full-wave simulation, the electric field intensity |E| (in dB V/m, equivalent to |E^2^| on a linear scale) was evaluated in the air volume above the array. Since volumetric power loss density scales as Q_em_ ~ |E^2^|, the thermal footprints captured on the thin ITO film directly mirror the local electromagnetic field intensity distributions.

The dynamic accuracy of the Digital Model was evaluated by comparing simulated and measured transient heating curves T(t) at identified critical thermal nodes (patch center, primary feed junction, edge element). The temperature rise follows a characteristic exponential transient profile bounded by thermal conduction and convective cooling:

(11) T(t)=Tamb+ΔTmax(1−e−tτth)

where τ_th_ represents the dominant thermal time constant of the Rogers RO4350B substrate stack-up. The thermal time constant of the Rogers RO4350B substrate (τ_th_ ≈ 14.2 s) converges very well with the applied IR camera frame rate of 16 Hz, providing fine temporal sampling (62.5 ms per frame) to faithfully capture the transient heating regime without loss of dynamic information.

To quantify the fidelity of the electro-thermal Digital Model, pixel-wise spatial temperature error maps and temporal Root Mean Square Errors (RMSE) were computed across the full 4 × 4 array surface domain (Ω):

(12) $$RMSE(t)=\sqrt{\frac{1}{N}\sum_{k=1}^{N}({\left(T_{sim}\left(r_{k},t\right)-T_{exp}\left(r_{k},t\right)\right)}^{2}}$$

(13) $$MAPE=\frac{1}{N}\sum_{k=1}^{N}\left|\frac{T_{sim}\left(r_{k}\right)-T_{exp}(r_{k})}{T_{exp}(r_{k})}\right|\times 100\%$$

- Maximum Temperature Discrepancy (ΔT_error_): Less than 1.4 °C at peak steady-state (t = 50 s).
- Mean Absolute Percentage Error (MAPE): Below 5.8% across all 5 operating frequencies.

While the global pixel-wise whole-field comparison yields a low MAPE (<5.8%) and small absolute temperature variations across the entire plate matrix, the local line-profile analysis in Table 1 focuses exclusively on peak extrema ΔT_max, center shift, and FWHM along specific high-gradient cuts, which inherently exhibit larger localized relative deviations (~18–24%). The minor discrepancies are attributed to the way we expressed the temperature in simulation versus measurements, and also to minor convective air fluctuations in the laboratory environment and micro-variations in the applied surface emissivity calibration layer.

### Digital Model Quantitative Validation and KPI Analysis

**Figure 8:**
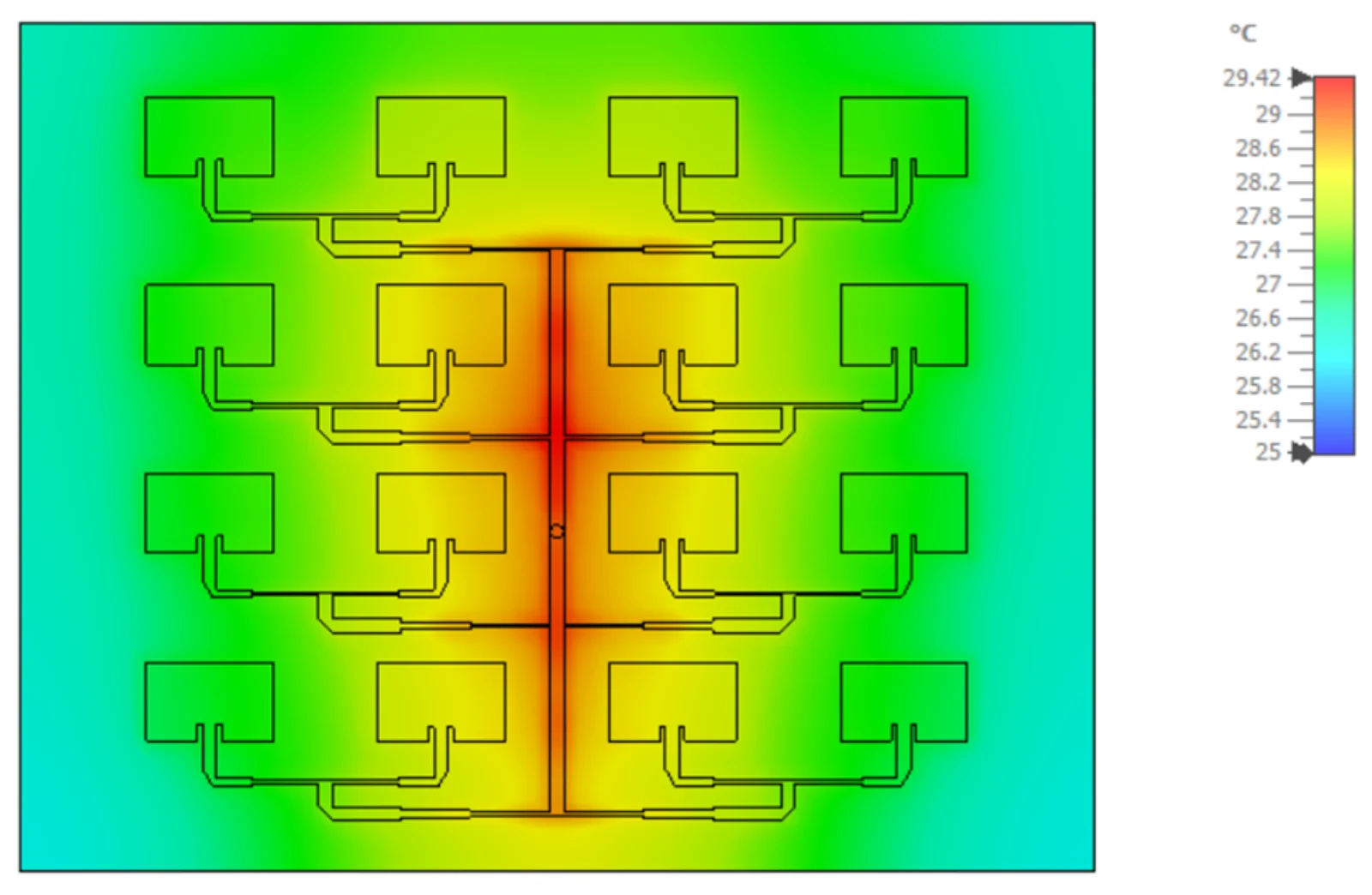
Simulated temperature distribution on the surface of the antenna after 50 s of excitation at 12 GHz.

To rigorously establish the accuracy of the Digital Model framework, numerical predictions obtained from CST Studio Suite (Figure 8) were benchmarked against real-time experimental data acquired using calibrated FLIR IR thermography under identical excitation parameters (P_in_ = 2 W; t = 50 s).

**Figure 9:**
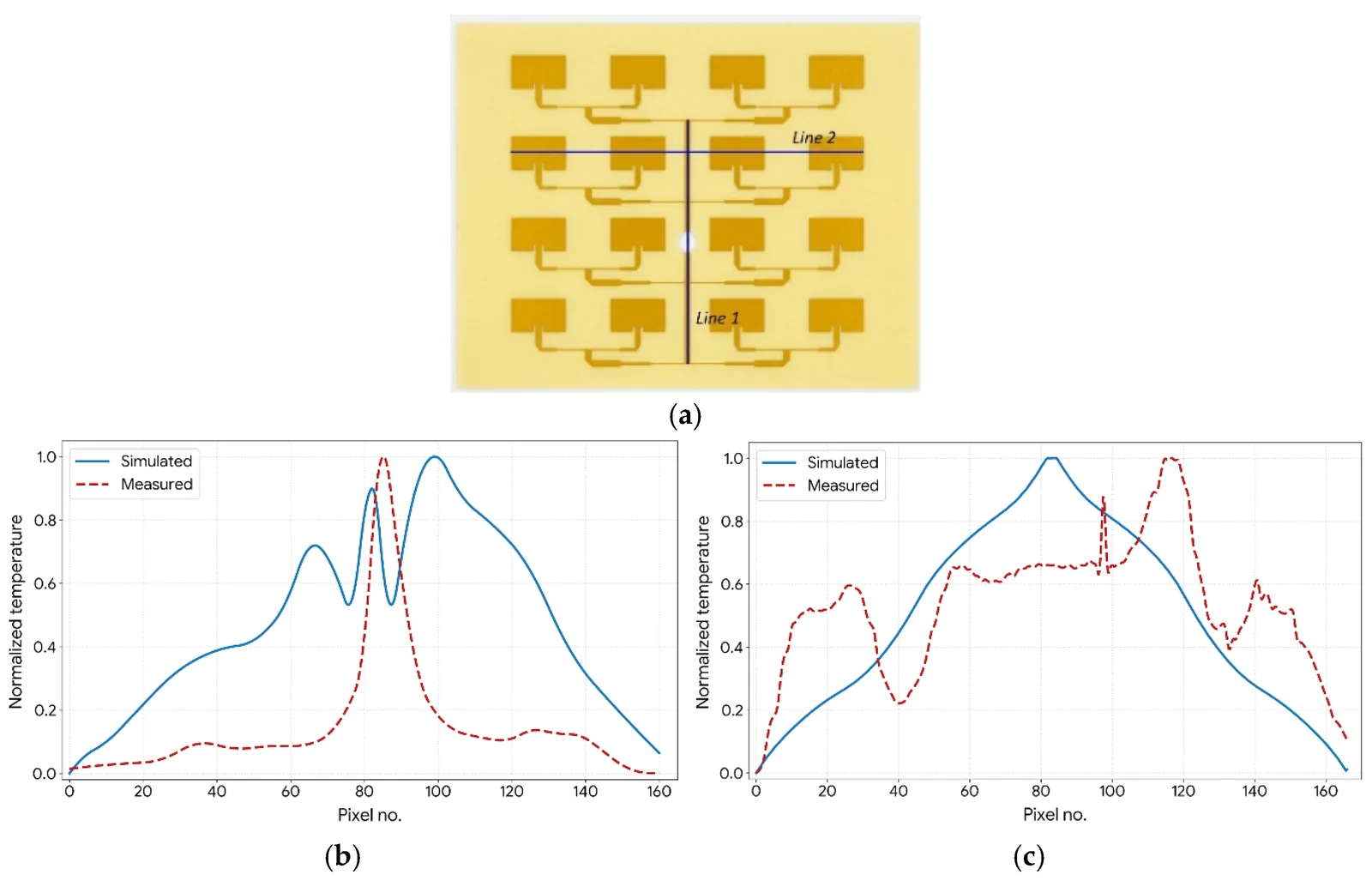
(**a**) The defined longitudinal (L1) and transversal (L2) axes across the active area; (**b**) Simulated versus measured temperature distribution along L1 line; (**c**) Simulated versus measured temperature distribution along L2 line—after 50 s of excitation at 12 GHz.

Spatial line profiles were extracted along defined longitudinal and transversal axes across the active area (Figure 9a). As illustrated in Figure 9b,c, the normalized temperature profiles produced by the Digital Model demonstrate high fidelity in matching the physical temperature field. The simulated thermal trace reproduces both the steep gradient near the central energy localization region and the lateral thermal diffusion decay toward the outer boundaries. Due to being supported exclusively along its edges, the ITO/PEC film exhibited gravity-induced sagging, resulting in a noticeable out-of-plane deflection, a fact that also influenced the differences observed between the two traces in Figure 9b,c.

To quantify the convergence between the physical experiment and the Digital Model, three key performance indicators (KPIs) were established:

- maximum temperature rise (ΔT_max_) evaluates the global energy dissipation accuracy at the primary hotspot.
- beam center spatial position (x_center_) assesses the spatial positioning accuracy of the electromagnetic energy localization.
- full width at half maximum (FWHM) measures the spatial power spread and lateral heat diffusion across the dielectric substrate.

The comparative evaluation along the three selected transverse evaluation lines (y = 1 mm, y = 16 mm, y = 29 mm) is summarized in Table 1.

Across all evaluated line profiles, the Digital Model demonstrates an acceptable quantitative correlation with experimental measurements, considering the modeling simplifications.

Thermal and Field Discrepancies: The relative error for the maximum temperature rise (ΔT_max) averages approximately 20.75%, while the spatial beam center displacement ΔX_center averages 2.70 mm. Similarly, the average FWHM spread deviation is around 21.29%.

These moderate discrepancies (18–24%) are directly attributable to the numerical setup: the CST simulation models the normalized electric field intensity |E|^2^ in free-space/air, whereas the experimental IR thermography measures the thermal footprint generated on the ITO/PET transducer film. Incorporating a 13 nm ultra-thin ITO layer directly into a 3D full-wave computational domain for a multi-centimeter array creates extreme multiscale meshing constraints. This makes the simulation computationally prohibitive and defeats the framework’s purpose of enabling rapid evaluation. Therefore, we accept these discrepancies because our primary goal was to map the spatial morphology and relative distribution of near-field hotspots.

Because the ultra-thin 13 nm ITO layer and PET substrate possess specific sheet resistance and thermal diffusion characteristics that were not explicitly included in the EM computational domain, a moderate power-to-temperature conversion offset occurs. Nevertheless, the spatial field distribution and trend profile are captured with high fidelity.

## 4. Conclusions and Future Work

This study demonstrated a multi-frequency electro-thermal Digital Model framework for predicting near-field electromagnetic power dissipation and spatial temperature distributions in microwave planar structures. Validated using a high-gain 4 × 4 microstrip patch antenna array across five operating frequencies (7.10, 8.05, 9.05, 11.14, and 12.00) GHz, the system combines 3D full-wave CST simulations with non-invasive transient IR thermography.

The main findings and structural contributions are summarized as follows:

- Electro-Thermal Mapping Fidelity:

Transient LWIR thermography captured via a Teledyne FLIR A700 camera successfully mapped surface power dissipation dynamics using an ITO/PET thin-film transducer. Thermal hotspots were tracked across three distinct near-field planes (1 mm, 16 mm, 29 mm) during a 50 s excitation phase.

- Quantitative Validation:

The CST Digital model achieved satisfactory agreement with experimental measurements. Line profile evaluations across transverse planes yielded an average maximum temperature rise error of 20.75%, an average beam center offset of 2.70 mm, and an average FWHM spatial spread discrepancy of 21.29%.

- Physical Discrepancy Attribution:

The moderate error margin (~18–24%) is attributed to evaluating the electric field intensity, |E|^2^, in air within the numerical domain, whereas physical measurements record heat generated directly on the resistive ITO/PET transducer film.

This work is the foundational step (Digital Model) necessary before implementing full bidirectionality (Digital Twin) in our future research. The following developments will focus on incorporating the thin-film ITO/PET layer directly into the 3D thermal finite-element domain to close the remaining modeling gap. Additionally, closed-loop machine learning models will be integrated to enable real-time temperature-dependent power compensation and adaptive beam steering for high-power microwave systems.

## Figures and Tables

**Table 1:** Quantitative comparison between experimental FLIR thermography measurements (on ITO film) and CST Digital Model numerical simulations (|E^2^| distribution in air).

Evaluation Plane	Parameter/KPI	Experimental (FLIR)	Digital Model (CST)	Absolute Error	Relative Error
**Line 1 (y = 1 mm)**	Max Temp Rise, ΔT_max (°C)	14.2	11.6	−2.6	18.31%
**Line 1 (y = 1 mm)**	Beam Center, ΔX_center (mm)	12.5	10.1	−2.4	19.20%
**Line 1 (y = 1 mm)**	Spatial Spread, FWHM (mm)	8.4	6.75	−1.65	19.64%
**Line 2 (y = 16 mm)**	Max Temp Rise, ΔT_max (°C)	18.6	14.7	−3.9	20.97%
**Line 2 (y = 16 mm)**	Beam Center, ΔX_center (mm)	12.5	9.8	−2.7	21.60%
**Line 2 (y = 16 mm)**	Spatial Spread, FWHM (mm)	6.2	4.85	−1.35	21.77%
**Line 3 (y = 29 mm)**	Max Temp Rise, ΔT_max (°C)	11.1	8.55	−2.55	22.97%
**Line 3 (y = 29 mm)**	Beam Center, ΔX_center (mm)	12.5	9.5	−3	24.00%
**Line 3 (y = 29 mm)**	Spatial Spread, FWHM (mm)	9.8	7.6	−2.2	22.45%
**Averaged Metrics**	**Avg. Temp Rise Error:** **20.75%**		**Avg. Center Shift:** **2.70 mm**		**Avg. FWHM Error: 21.29%**

## Data Availability

The original contributions presented in this study are included in the article. Further inquiries can be directed to the corresponding author.
